# Integrating multiple slack bus operations and metaheuristic techniques for power flow optimization

**DOI:** 10.1038/s41598-025-01393-5

**Published:** 2025-05-14

**Authors:** Swaechchha Dahal, Gunne John Hegglid, Jonas Kristiansen, Bhupendra Bimal Chhetri, Sambeet Mishra, Thomas Øyvang

**Affiliations:** 1 Department of Electrical, IT and Cybernetics, University of South Eastern Norway, Porsgrunn, Norway; 2 Department of Electrical and Electronics Engineering, Kathmandu University, Dhulikhel, Nepal; 3 Department of Electric Energy, Norwegian University of Science and Technology, Trondheim, Norway

**Keywords:** Cuckoo search algorithm, Grey wolf optimization, Metaheuristic algorithms, Multiple slack bus operation, Optimal power flow, Particle swarm optimization, Reduced Nordic 44 model, Power loss minimization, Line loading optimization, Power system efficiency, Electrical and electronic engineering, Energy grids and networks

## Abstract

The increasing complexity of modern energy grids amplifies the importance of realistic power flow studies in power system analysis. This study implements a Multiple Slack Bus Operation (MSO) framework to enhance the realism and efficiency of optimal power flow (OPF) analysis. This paper introduces a comparative evaluation of three metaheuristic algorithms: particle swarm optimization (PSO), cuckoo search algorithm (CSA), and grey wolf optimization (GWO) within the MSO framework. These algorithms are assessed based on their effectiveness in minimizing system loss, optimizing line loading, adjusting the angle of the generator voltage, and optimizing the generation distribution. Using the Reduced Nordic 44 model and the IEEE benchmark test systems in various load conditions, the findings reveal that the GWO algorithm, when integrated with the MSO framework, achieves the most significant reduction in total system losses. The implementation of MSO alone reduced system losses by 5%, and its combination with GWO led to an additional 8.3% decrease. This study investigates the application of metaheuristic algorithms within a multiple slack bus context, highlighting their potential to enhance power network efficiency and suggesting broader applications for future power flow optimization strategies.

## Introduction

Power system operations have become more sophisticated due to the rapid development of modern energy networks, characterized by rising demand, the integration of renewable energy sources, and the adoption of smart grid technology. One of the significant challenges in power systems is to optimize the power flow between different pricing areas^1^. The Nordic power system is a synchronous system divided into different pricing areas with interconnected grids spanning across several Nordic countries and is known for its strong interconnection and power trading system^2^. Efficient power flow between different pricing areas is a critical challenge, as imbalances can lead to increased transmission losses, congestion, and thus, elevated electricity prices and reduced system reliability^3,4^.

Optimal power flow (OPF) studies play a crucial role in ensuring the efficient, reliable, and economical operation of these complex power systems. By determining the optimal operating conditions, OPF helps minimize generation costs, reduce transmission losses, and enhance system stability^5,6^. The determination of OPF intertwines power systems and operations research, presenting theoretical and practical challenges^7^. The OPF problem has been solved using a variety of optimization techniques due to its complexity and significance such as classical, deterministic algorithms, metaheuristic algorithms (e.g., swarm-inspired, human-inspired, evolutionary-inspired, physics-inspired), and artificial neural network (ANN)^7,8^. The metaheuristic algorithms have been employed extensively to address OPF issues and have gained traction due to their efficacy in large-scale optimization^5,9,10^. The choice of metaheuristic algorithms for solving the OPF problem merits discussion, particularly when compared to linearization approaches using Mixed Integer Linear Programming (MILP) solvers. While linearization techniques like DC power flow approximations can provide global optima for simplified models, they inherently sacrifice accuracy by neglecting voltage variations and reactive power flows^11^, which are crucial in large interconnected systems. The AC power flow equations contain nonlinear terms including trigonometric functions and voltage products that make exact linearization challenging without substantial approximation errors. Metaheuristic algorithms offer several advantages in this context^12^.

The integration of renewable energy sources introduces additional challenges to power system optimization, making advanced methods increasingly important^13^. proposed a homomorphic encryption-based approach for resilient distributed energy management under cyber-attacks, highlighting security concerns in modern power systems. For multi-objective optimization in microgrids^14^, developed a penalty-based boundary intersection approach using deep reinforcement elite learning to simultaneously optimize economic cost, voltage deviation, and frequency stability^15^. provided a comprehensive review of decentralized control structures for microgrids, emphasizing their advantages in reliability and flexibility compared to centralized approaches. For multi-energy systems^16^, presented a collaborative planning framework integrating complete hydrogen energy chains, demonstrating significant reductions in renewable energy curtailment^17^. proposed an initialization-free distributed algorithm for economic dispatch problems, addressing the limitations of traditional distributed optimization methods. These studies underscore the growing complexity of power system optimization and the need for advanced techniques like an integrated MSO-metaheuristic approach to address challenges in modern energy systems with high renewable penetration.

This paper extends to paper^18^ where three different metaheuristic algorithms- Particle Swarm Optimization (PSO), Cuckoo Search Algorithm (CSA), and Grey Wolf Optimization (GWO)-are compared. These particular algorithms were selected based on their distinct developmental time frames, which illustrate the flexibility and advancement of optimization methods across time. Numerous studies and developments, such as hybridization approaches, demonstrate their adaptability in a variety of optimization scenarios. The “No Free Lunch” theorem (NFL) asserts that no single algorithm is inherently better than any other for every optimization issue^19^; yet, it also emphasizes that algorithmic efficacy is inextricably linked to the particulars of the problem being solved. Thus, this selection represents a balanced mix of diversity and shows efficiency, aiming for a holistic evaluation. The initial study provided a balanced evaluation of these algorithms, demonstrating their adaptability in various optimization scenarios.

Building upon that foundation, this extended study introduces a Multiple Slack Bus Operation (MSO) framework to enhance the realism and efficiency of OPF analysis in complex power systems. MSO is a pivotal concept in the realm of power flow optimization, which is especially crucial in systems like the Nordic grid. Instead of relying on a single slack bus to balance the system, this method distributes the total system imbalance across several generators. This distributed balance is often related to a deviation from the load-frequency control mechanism, with these generators generating based on participation factors^20,21^. The conventional power flow formulation, reliant on a single slack bus model, may not yield accurate results for minimizing generation cost and power losses^22^. MSO can offer more flexibility in power flow management and potentially enhance system efficiency^20^, which is especially beneficial in complex, interconnected power systems.

To contextualize the research within the existing body of knowledge, Table 1provides an overview of state-of-the-art methodologies in metaheuristics and multiple slack bus operations. This summary highlights recent studies, their aims, methods, key findings, and their relevance to this study. Recent advances in optimization techniques across various domains offer valuable insights for power system optimization problems^23^. proposed a metalearning-based alternating minimization algorithm for nonconvex optimization that demonstrates superior adaptability for solving complex problems^24^. developed a physics-informed network approach for parameter identification that maintains high explainability while achieving excellent performance. In transportation logistics, Xu and^25^addressed complex optimization with multiple constraints through adaptive algorithms with similarities to power system challenges^26^. demonstrated the benefits of cooperative optimization for electric bus networks, showing how coordinated scheduling can significantly reduce operational costs. In maintenance optimization^27^, proposed an adaptive maintenance window approach considering both reliability and cost objectives, similar to the considerations in power flow optimization in this study. These diverse optimization approaches highlight the importance of adaptive, problem-specific strategies when dealing with complex systems, validating the approach of comparing multiple metaheuristic techniques for the MSO framework.

**Table 1:** Overview of State-of-the-Art Methodologies in Metaheuristics and Multiple Slack Bus Operation.

**Article(s) & Year**	**Aim**	**Methods**	**Key Findings**	**Relevance to this study**
^21^ - 2020	To formalize the distributed slack bus formulation using participation factors and nominal injections for accurate power flow solutions.	Distributed slack bus model using economic dispatch setpoints and AGC participation factors.	Demonstrated that the distributed slack bus formulation yields more accurate estimates of bus voltage magnitudes and phase angles, offering a realistic representation of generator dynamics compared to the single slack bus model.	Highlights the importance of realistic generator dynamics in power flow analysis.
^22^ - 2022	To propose a cooperative game theory-based model to compute participation factors for distributed slack buses.	Cooperative game theory and Shapley value for power flow analysis.	Achieved reduction in generation cost and power losses; effective allocation of slack power based on generator capabilities.	Supports the use of distributed slack bus for improved efficiency.
^28^ - 2023	To investigate the effect of slack bus selection on power losses and power flows in robust and weak grids.	Load flow analysis using Newton-Raphson and Gauss-Seidel methods on the IEEE 14-bus test system.	Found that slack bus selection does not significantly affect system losses in robust grids but has notable effects in weak grids; highlights the importance of considering grid strength in slack bus configuration.	Emphasizes the critical role of slack bus configuration in different grid conditions.
^29^ - 2023	To develop a novel algorithm for computing multi-area power flows suitable for interconnected AC synchronous areas.	Iterative matrix-based composition/decomposition approaches; compared with software like DIgSILENT PowerFactory.	Found that treating areas independently improves computational efficiency in multi-area power flow problems; highlights the potential of multi-slack bus methodologies.	Aligns with the study’s emphasis on multi-area power flow and multiple slack bus formulations in large interconnected systems.
^30^- 2024, ^31^- 2024	To incorporate transmission loss into LMP calculations while ensuring fairness in slack bus allocation and improving robustness; to develop load flow analysis methods suitable for islanded microgrids considering frequency variations.	DC Optimal Power Flow (DCOPF), Second-Order Cone Programming (SOCP), and duality analysis with distributed slack bus; modified decoupled Newton-Raphson method.	Developed a load-weighted distributed slack bus approach to ensure fair slack allocation; introduced a convexified DCOPF using SOCP for efficient solutions; improved convergence in load flow calculations for islanded microgrids incorporating frequency variations.	Relevant to the concept of multiple slack bus operation and fairness in slack allocation.
^32^ - 2012	To develop a distributed slack bus algorithm for economic load dispatch (ELD), aiming to reduce system generation costs while distributing system losses effectively.	Newton-Raphson method with participation factors and the distributed slack bus concept.	Achieved reduction in generation costs by distributing the slack bus burden across multiple generators; system losses remained the same.	Demonstrates the benefit of distributed slack bus operation in reducing generation costs.
^33^ - 2022	To provide a comprehensive overview of state-of-the-art optimal power flow (OPF) techniques, focusing on emerging research directions and applications.	Survey and review, focusing on OPF methodologies including decomposition, decentralized optimization, and convex relaxation techniques.	Identified key advances in distributed optimization, stochastic techniques for managing uncertainty, and novel conic relaxation methods for improved solution robustness.	Provides insights into advanced OPF methods, such as decentralized optimization, which relate to the focus on applying metaheuristic algorithms for power flow optimization across multi-area pricing regions.
^10^- 2021, ^34^- 2023	To review the use of metaheuristic optimization methods for OPF, scheduling, and planning tasks in smart grids.	Metaheuristic algorithms like Genetic Algorithms (GA), Particle Swarm Optimization (PSO), Ant Colony Optimization (ACO), Differential Evolution (DE), Swarm Intelligence (SI), and Artificial Bee Colony (ABC).	Highlighted the broad applicability and effectiveness of metaheuristics in addressing non-convexity, multi-objectivity, and discrete variables in smart grids; identified ABC as particularly effective due to its fast convergence and computational efficiency.	Provides foundational understanding of metaheuristic techniques used in OPF.
^35^- 2024, ^36^- 2023	To minimize energy losses in power distribution systems and optimize power flow using advanced metaheuristic algorithms; to evaluate performance of different metaheuristic techniques using benchmark test functions.	Advanced metaheuristic algorithms and the ladder iterative technique within an EMS.	Achieved significant energy savings (e.g., 18% reduction); algorithms like Artificial Ecosystem Optimization (AEO) demonstrated superior performance; provided insights into performance evaluation and algorithm effectiveness.	Supports the use of advanced metaheuristics for loss minimization and efficient power flow control; helps assess strengths and weaknesses of optimization techniques.
^37^ - 2024	To critically review recent metaheuristics, highlight trends, and identify issues with the proliferation of new optimization algorithms.	Reviewed 111 studies on recent metaheuristics, analyzing their motivations, approaches, and innovations.	Found that most new metaheuristics lack true innovation, often being slight modifications of existing methods; suggested a need for stricter standards to identify genuinely novel contributions.	Reinforces the ongoing challenges in metaheuristic development, highlighting the relevance of using well-established methods in realistic settings.
^38^- 2024, ^39^- 2024	To solve the Security-Constrained Optimal Power Flow (SCOPF) using metaheuristic algorithms under line outage scenarios; to review various metaheuristic algorithms for OPF optimization, focusing on hybrid systems.	Binary GA and PSO on IEEE test systems; metaheuristic algorithms like GA, PSO, SA, and hybrid approaches.	Found that Binary GA achieved better loss minimization but was computationally expensive; PSO achieved lower fuel costs; provided a comprehensive evaluation of different algorithms, highlighting their strengths and weaknesses in solving complex OPF problems.	Highlights the application of metaheuristics in SCOPF, emphasizing their role in managing contingencies effectively, which is pertinent to optimizing power flow in interconnected systems; offers valuable insights into various metaheuristic techniques.

### Research gap and contributions

Despite the significant advancements in both metaheuristic optimization techniques and multiple slack bus operation methodologies, there remains a notable gap at their intersection. As highlighted in Table 1, numerous studies have explored the effectiveness of metaheuristic algorithms in solving OPF problems. For instance^10,34^, reviewed the broad applicability of various metaheuristic algorithms, emphasizing their effectiveness in addressing non-convexity and multi-objectivity in smart grids^35,36^. demonstrated significant energy savings using advanced metaheuristic algorithms, underscoring their potential in loss minimization and efficient power flow control.

Similarly, research focusing on multiple slack bus operations has shed light on its benefits in power flow analysis^21^. formalized the distributed slack bus formulation using participation factors, revealing more accurate estimates of bus voltage magnitudes and phase angles compared to the single slack bus model. Similarly, other studies also highlight the importance of realistic generator dynamics and effective slack power allocation in enhancing system efficiency.

However, there is a lack of studies that integrate metaheuristic optimization techniques within an MSO framework. While the individual merits of metaheuristic algorithms and MSO have been established, their combined potential remains under-explored. Multiple slack bus operation provides a more realistic and flexible representation of power systems, especially in complex and interconnected networks where relying on a single slack bus is insufficient. On the other hand, metaheuristic algorithms are powerful tools for solving complex optimization problems like OPF, but their performance can vary depending on the specific system and context. Therefore, comparing different metaheuristic methods within an MSO framework is essential in identifying the most effective algorithm for this realistic power system model. This gap presents an opportunity to bridge the domains of metaheuristic optimization and multiple slack bus operation. By integrating well-established metaheuristic algorithms within the MSO framework and applying them to realistic power system models, this research enhances the accuracy and efficiency of OPF analysis by demonstrating tangible improvements in power system performance-specifically a 5% reduction in system losses from MSO implementation alone, with an additional 8.3% decrease when combined with GWO, ultimately contributing to improved power network performance.

Building upon the identified research gap, the primary objective of this study is to integrate well-established metaheuristic algorithms-PSO, CSA, and GWO-within an MSO framework for optimal power flow optimization. By uniquely integrating metaheuristic techniques with the MSO framework and applying this novel approach to both IEEE benchmark test systems and the Reduced Nordic 44 model, this research makes significant contributions to power system optimization through:

1. Evaluating the effectiveness of each algorithm in minimizing system losses within the MSO framework.
2. Analyzing the impact of multiple slack bus configurations on power flow optimization and system efficiency.
3. Comparing the performance of metaheuristic algorithms in terms of convergence speed and solution quality.
4. Providing practical insights into the applicability of these algorithms in real-world power systems, highlighting potential challenges and benefits.

By bridging these gaps between metaheuristic optimization techniques and multiple slack bus operations, this research advances the field of OPF studies and provides practical solutions for modern energy systems. This integration can serve as a foundation for future studies aiming to optimize power flow in increasingly complex and interconnected power networks.

### Article organization

The subsequent sections of the paper is organized as follows: Section 3 establishes the mathematical model for the formulation of the OPF problem. Then, Section 4 describes the investigated metaheuristic algorithms within the MSO framework. Their implementation in different test systems is described in Section 5. Section 6 focuses specifically on the reduced Nordic 44 model as an application case. Finally, Section 7 demonstrates the integration of metaheuristics within the MSO framework before Section 8 concludes and suggests future research avenues.

## Optimal power flow problem formulation

The goal of classical OPF is to determine the most optimal settings for control variables in a power system, aiming to minimize a specific objective, such as cost or power loss, while also ensuring that certain technical constraints-both equality and inequality-are satisfied. The mathematical conceptualization of OPF goes back to^40^. Generally, an OPF problem can be formulated as follows:

1 $$\begin{aligned} \begin{aligned} \min _{x,u} \quad&F(x,u),\\ \text {subject to} \quad&g(x,u)=0,\\&h(x,u) \le 0, \end{aligned} \end{aligned}$$

where $F\mathbf {(x,u)}$ is the optimization objective, $g\mathbf {(x,u)}$ and $h\mathbf {(x,u)}$ represent equality and inequality constraints respectively, $\textbf{x}$ and $\textbf{u}$ represents control and state variables, respectively. Control variables are variables that the system operator can adjust to achieve the desired state variables^41^. Examples of control variables in the OPF problem include the generator set points, transformer tap ratios, and the use of shunt capacitors or reactors. Similarly, state variables are variables that represent the internal state of the power system and are used to describe the behavior of the system over time, such as the rotor angles and speeds of the generators, the voltages, and currents in the transmission lines, generator output powers.

### Constraints

It is important to consider several power flow limitations, including inequality and equality constraints, when maximizing the OPF goal. The OPF problem’s equality constraint, which assures that the active power produced by the power system’s generators equals the active load demands and active power losses, results from the balance between power input and output. Reactive power adheres to the same balance principle. Therefore, the equality constraints can be formulated as follows^33^:

2 $$\begin{aligned} P{_i}^{G} =P{_i}^{L} + V{_i} \sum _{k=1}^{N} V_k \Big ( G_{ik} cos(\delta _i - \delta _k) + B_{ik} (sin(\delta _i - \delta _k) \Big ), \forall _i \in N, \end{aligned}$$

3 $$\begin{aligned} Q{_i}^{G} =Q{_i}^{L} + V{_i} \sum _{k=1}^{N} V_k \Big ( G_{ik} sin(\delta _i - \delta _k) - B_{ik} (cos(\delta _i - \delta _k) \Big ), \forall _i \in N, \end{aligned}$$

where, $P{_i}^{G}$, $Q{_i}^{G}$ represent active and reactive power generation and $P{_i}^{L}$, $Q{_i}^{L}$ represent active and reactive load demand respectively. $G_{ik}$ and $B_{ik}$ represent line conductance and susceptance between bus $\textbf{i}$ and $\textbf{k}$ respectively.

OPF inequality constraints result from considerations of operating limits, such as the power flows through transmission lines, generation levels of individual generators, limits on voltage magnitudes on buses, and limits on the reactive power output of generators and reactive devices like capacitors and reactors. The inequality constraints used in the study are as follows^42^:

4 $$\begin{aligned} P{_i}^{G,min} \le P{_i}^{G} \le P{_i}^{G,max} \hspace{25pt} \forall _i \in G \end{aligned}$$

5 $$\begin{aligned} Q{_i}^{G,min} \le Q{_i}^{G} \le Q{_i}^{G,max} \hspace{25pt} \forall _i \in G \end{aligned}$$

6 $$\begin{aligned} V{_i}^{min} \le V{_i} \le V{_i}^{max} \hspace{25pt} \forall _i \in N \end{aligned}$$

### Optimization objective

The objective of OPF formulation in this study is to minimize the real power loss in the system. The problem can be formulated as follows:

7 $$\begin{aligned} P_{loss} = \sum _{i=1}^{NL} \sum _{k\ne i}^{NL} G_{ik} \Big (V{_i}^{2}+ V{_k}^{2} - 2V{_i}V{_k} cos(\delta _i - \delta _k), \Big ) \end{aligned}$$

where $V_i$ is the voltage magnitude at bus i, $G_{ik}$ is the conductance of line $\mathbf {i-k}$, $\delta _i$ is the voltage angle at bus i, and $\textbf{NL}$ is the number of transmission lines in the system.

In this implementation, the control variables (x) primarily consist of generator active power outputs, while state variables (u) include bus voltage magnitudes and angles. The objective function represents the total active power loss in the system as formulated in Equation 7. The equality constraints ensure power balance at each bus as expressed in Equations 2 and 3, where active and reactive power generation must equal the sum of load demands and power flows through connected lines. The inequality constraints include the operational limits on generator outputs and bus voltages as defined in Equations 4–6, ensuring that all components operate within their safe technical boundaries. This formulation allows us to find the optimal operating point that minimizes system losses while maintaining all operational constraints within their respective limits.

### Multiple slack bus formulation

A realistic depiction of power systems does not rest upon a single slack bus assumption. Envisioning a single bus to compensate for system losses, especially in vast networks, is overly simplistic. To address this limitation, the concept of a multiple slack bus, where power deficits are distributed among various generator buses, emerges as a practical alternative^20^.

The participation factor, a weight designated to each generator bus, is pivotal in this distributed paradigm^32^. It ensures the power allocation aligns with individual generator’s capacity limits. While determining these weights, various aspects are considered, like the capacity of individual generators, their distance from demand centers, interconnection strengths, and dependencies from other grid systems. In this work, the participation factor is grounded in generator capacities. The capacity of a generator indicates its efficiency and associated losses. As the generator’s size increases, its efficiency tends to improve for a given consistent loss. As loads are allocated across generators, they approach their maximum generation thresholds. A higher capacity generator offers a wider buffer before reaching this threshold. As the efficiency drops when generators approach their maximum limits, prioritizing larger generators becomes beneficial.

In this study, the participation factor is derived from the real-time active power output of the system’s generators. After each iteration, the power flow determines the individual active power produced by each generator, aligned with the OPF framework. It’s crucial to note that the generation becomes optimal at this specific active power value rather than its initial set capacity. Therefore, when distributing the deficit loss across the generators, this instantaneous active power output serves as a model for the participation factor, $K_i$^32,43^.

8 $$\begin{aligned} K{_i}=\frac{P{_i}^{G}}{\sum _{i=1}^{N} P{_i}^{G}} \end{aligned}$$

Now, incorporating the consideration for the multiple slack bus system where each generator compensates for its share of losses, the active power balance equation is modified. Given the total power loss in the system as $P_{loss}$ and the participation factor for the $i_{th}$ generator as $k_i$, the additional power that the $i_{th}$ generator needs to produce to compensate for its share of the system loss is:

9 $$\begin{aligned} \Delta {P{_i}^{G}} = K{_i} \times {P{_{loss}}}. \end{aligned}$$

Adjusting the earlier active power equation (2) to account for this additional power from each generator:

10 $$\begin{aligned} P{_i}^{G,new} = P{_i}^{L} + \Delta P{_i}^{G} \end{aligned}$$

Equation (10) ensures that the total generation meets the total demand plus losses, with each generator contributing based on its participation factor. The reactive power of the buses remains the same.

## Optimal power flow solution with metaheuristic algorithms within the MSO framework

**Fig. 1:**
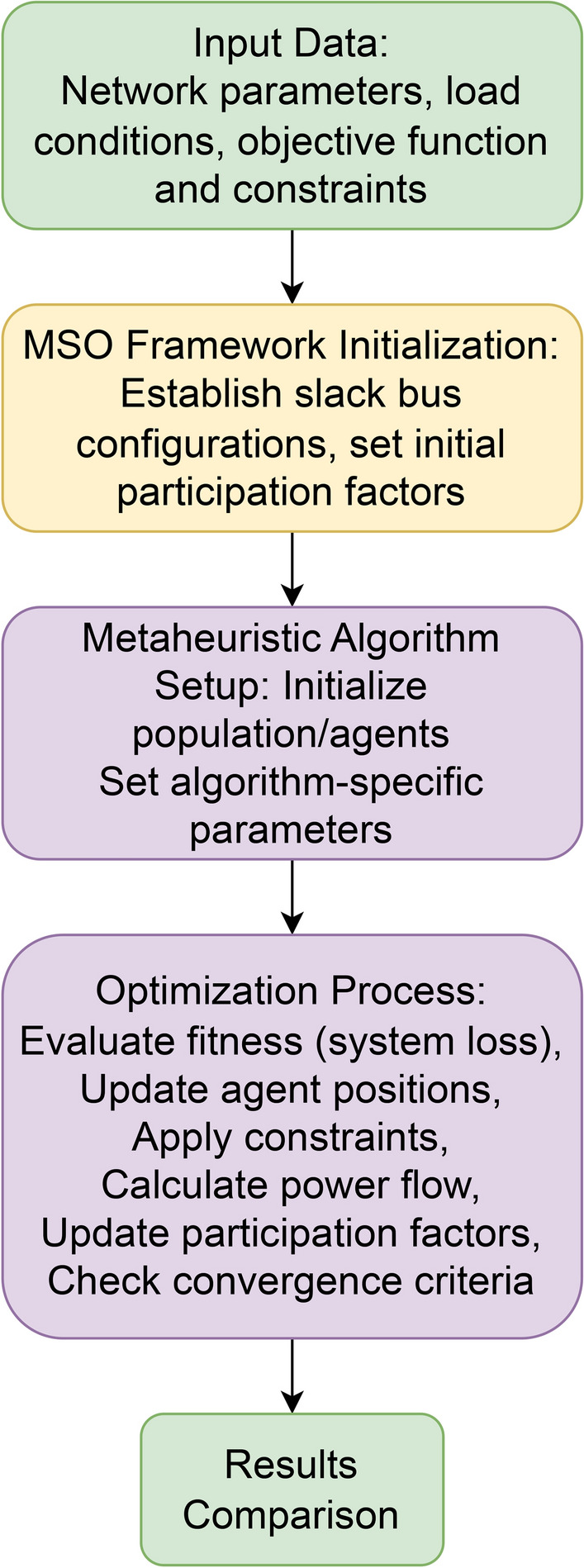
Flowchart of the integrated MSO-metaheuristic approach for optimal power flow.

This section delves into the implementation of three well-established metaheuristic algorithms-Particle Swarm Optimization (PSO), Cuckoo Search Algorithm (CSA), and Grey Wolf Optimization (GWO)-within the Multiple Slack Bus Operation (MSO) framework. Each algorithm is adapted to accommodate the distributed slack bus configuration, ensuring that the optimization process reflects the realistic dynamics of complex power systems. Figure 1 shows the overall workflow of the integrated approach, combining the MSO framework with metaheuristic optimization techniques to solve the OPF problem. The selection of PSO, CSA, and GWO algorithms for this study is based on several key considerations. These algorithms represent different developmental periods in metaheuristic optimization, with PSO being one of the earlier widely-adopted techniques, CSA representing a mid-period development, and GWO being a more recent innovation. This diversity allows to evaluate how algorithmic advancements translate to performance improvements in power system optimization. Additionally, these algorithms employ fundamentally different search mechanisms-PSO relies on swarm intelligence with social and cognitive learning components, CSA utilizes Lévy flights for exploration, and GWO employs a hierarchical leadership structure-providing a comprehensive basis for comparison. The MSO framework is selected because it offers a more realistic representation of actual power system operations compared to traditional single slack bus approaches, particularly in interconnected systems where multiple generators contribute to system balance. A detailed explanation of the theoretical foundations, adaptations for MSO, implementation specifics, and practical considerations for each algorithm is provided.

### Particle Swarm Optimization (PSO) within the MSO framework

PSO is a popular metaheuristic algorithm inspired by how social animals, like birds, behave when flocking. In PSO, a group of particles, each representing a potential solution, moves through the problem’s solution space. Each particle adjusts its position based on both its own best experience and the best solution discovered by neighboring particles. Through this process, the algorithm continuously updates the movement and position of the particles to find optimal solutions^44^. The position, $\textbf{x}$, and velocity, $\textbf{v}$ of individual particle, $\textbf{i}$, in a $\textbf{n}$-dimensional search space can be represented by following equations^45^.

11 $$\begin{aligned} x{_i}^{k+1} = x{_i}^{k} + v{_i}^{k+1} \end{aligned}$$

12 $$\begin{aligned} v{_i}^{k+1} = v{_i}^{k} + \alpha _i (x{_i}^{lbest} - x{_i}^{k}) + \beta _i (x^{gbest} - x{_i}^{k}) \end{aligned}$$

where

$x{_i}^{k}$ is the particle position at iteration $\textbf{k}$

$v{_i}^{k}$ is the velocity of particle at iteration $\textbf{k}$

$\alpha _i$, $\beta _i$ are random numbers between 0 and 1

$x{_i}^{lbest}$ is the local best of individual $\textbf{i}$

$x^{gbest}$ is the global best of the swarm

For the adaptation of PSO within the MSO framework, this study modifies the objective function and constraints to incorporate multiple slack buses. Generator participation factors are integrated into the optimization process, influencing the particles’ movement within the search space. The fitness function evaluates both power loss and adherence to participation factors, ensuring a realistic and balanced distribution of slack power among the generators.

### Cuckoo Search Algorithm (CSA) within the MSO framework

CSA is another metaheuristic optimization technique commonly applied in power system problems. It is inspired by the unique nesting behavior of cuckoo birds, which lay their eggs in the nests of other birds, leaving the host birds to raise their offspring. In CSA, each candidate solution is representes the cuckoo egg, and the quality of the solution is measured by the objective function, much like the quality of the egg. The algorithm works through three key steps: randomly selecting a host nest, laying a cuckoo egg (new solution), and having the host bird either accept or reject the egg based on its quality. By following this process, the population of candidate solutions is continuously updated by replacing lower-quality solutions with new ones. The concept was first introduced by Yang and Deb in 2009^46^.

CSA employs Lévy flights to explore the search space, enabling a balance between local and global search capabilities. The algorithm begins by defining the objective function and constraints, followed by randomly initializing a population of nests, each representing a possible solution within the search space of control variables. A maximum number of iterations is set, and the fitness of each nest is evaluated based on the objective function. The nests are then sorted in ascending order of fitness. A random nest is chosen to act as the “cuckoo.” A new solution, or “cuckoo’s egg,” is generated using a random walk or Levy flight within the search space. If the new solution is better than the current one and meets a certain probability threshold, it replaces the current nest’s solution. A random walk is also applied to further explore the search space, ensuring that new solutions stay within the control variable bounds. This process repeats until a stopping criterion, such as the maximum number of iterations, is reached. Finally, the best solution found by the algorithm is returned.

To adapt CSA for the MSO framework, this research modifies the fitness function to account for distributed slack bus considerations, similar to the approach used for PSO. The participation factors are integrated into the evaluation of each nest (solution), ensuring that the generated solutions respect the distributed slack responsibilities. The fitness function evaluates both power losses and deviations from the desired participation factors, penalizing solutions that fail to appropriately distribute slack power among the generators.

### Grey Wolf Optimization (GWO) within the MSO framework

GWO is a relatively new metaheuristic algorithm that has gained traction for solving power system optimization problems. It is modeled after the hunting behavior of grey wolves. In this algorithm, the population of candidate solutions is representative of a pack of wolves, with each wolf symbolizing a potential solution to the problem. The algorithm works by iteratively updating the position of each wolf within the solution space, guided by the leadership hierarchy in the pack. The alpha wolf represents the best solution so far, the beta wolf holds the second-best solution, the delta wolf represents the third-best, and the omega wolf is a randomly selected solution from the group. Introduced by Mirjalili in 2014, GWO has been widely studied for its effectiveness in various optimization scenarios^47^.

The process begins by defining the objective function and constraints that guide the optimization problem. A population of wolves is then randomly initialized within the search space of control variables. The algorithm sets a maximum number of iterations for convergence. Each wolf’s fitness is calculated by evaluating the objective function, and the top three wolves-alpha, beta, and delta-are identified based on their fitness levels. The positions of the wolves are updated using specific equations. For the alpha wolf, the position is updated as in Equation 13.

13 $$\begin{aligned} X_1 = X_{\alpha } - A * D_{\alpha } \end{aligned}$$

Similarly, for the beta and delta wolves, the positions are updated using Equation 14 and 15.

14 $$\begin{aligned} X_2 = X_{\beta } - A * D_{\beta } \end{aligned}$$

15 $$\begin{aligned} X_3 = X_{\delta } - A * D_{\delta } \end{aligned}$$

The rest of the wolves update their positions by averaging the positions of the top three wolves as shown in Equation 16:

16 $$\begin{aligned} X_i = \frac{X_1 + X_2 + X_3}{3} \end{aligned}$$

where, A is the current search agent position, and D is the distance between the current agent and the alpha, beta, or delta wolf.

These updated positions are checked against boundary constraints to ensure they remain within the allowable limits of the decision variables. The fitness of each updated position is then re-evaluated. This process continues iteratively until the stopping criterion, usually a maximum number of iterations, is met. The best solution found, represented by the position of the alpha wolf with the lowest fitness value, is returned as the final solution.

The algorithm fits well with the MSO framework due to its hierarchical structure, which mirrors the distributed control of generators. In this approach, the alpha, beta, and delta wolves represent the key generators with the largest participation factors, steering the optimization process to efficiently distribute slack power. The fitness function evaluates both power losses and how well the solution adheres to the participation factors, ensuring that the optimization meets the requirements of the MSO framework.

In this study, each of these algorithms is applied to optimize the control variable, which is the real power output of the generators, to minimize power losses across the transmission lines. These losses are represented by the total real power loss in all the network’s transmission lines. The algorithms also account for key constraints, such as power balance, generator capacity limits, and bus voltage limits. By adhering to these constraints while minimizing the objective function, the metaheuristic algorithms can explore the complex, multi-dimensional solution space to identify the optimal configurations for solving the OPF problem.

## Comparison of Metaheuristic techniques for optimal power flow in various power systems

The performance of metaheuristic algorithms in solving OPF problems can vary significantly depending on the power system’s size, complexity, and configuration. To comprehensively evaluate the effectiveness of chosen algorithms, tests were conducted for comparison across multiple power system test cases. The test cases considered in this study include the IEEE 14-bus^48^, 39-bus^49^, and 118-bus systems^50^, as well as the Reduced Nordic 44 system (explained in Section 6), which represents a more complex and interconnected power network. Each system presents unique challenges in terms of power flow optimization, ranging from smaller, simpler networks to larger, more complex configurations and significant inter-area power transfers. By comparing these algorithms across varying levels of complexity, the aim is to determine which method offers the best trade-off between convergence speed and optimization accuracy in real-world scenarios.

### Computational experiments

The computational experiments for comparing the metaheuristic algorithms were conducted on an HP ZBook Fury laptop, equipped with an 11 th Gen Intel^®^ Core^TM^ i9-11950H processor and 32 GB of RAM. The algorithms were implemented using the Python programming language, leveraging the Pandapower library^51^ for power system simulations and optimization tasks.

Each algorithm was coded using standard optimization frameworks and was tested across the IEEE 14-bus, 39-bus, and 118-bus systems, and the Reduced Nordic 44 model under various load conditions. The computational resources provided sufficient capacity to handle the complexity and scale of these systems without significant performance bottlenecks.

The added complexity of the MSO-metaheuristic approach is justified through several key considerations. It provides a more realistic model of modern power systems where multiple generators share balancing responsibilities. The significant improvements in system efficiency and line loading profiles deliver tangible economic and operational benefits that outweigh the implementation complexity. Also, the approach’s adaptability across different test systems of varying sizes demonstrates its practical scalability for real-world applications, making it valuable for power system operators seeking more accurate and efficient optimization solutions.

To ensure a fair comparison between the three metaheuristic algorithms, all methods were evaluated under identical computational conditions. Each algorithm was implemented with a population size of 50 and executed for 500 iterations, resulting in exactly 25,000 function evaluations per algorithm across all test cases. This equal allocation of computational resources ensures that performance differences reflect the inherent capabilities of each algorithm rather than variations in implementation. The experiments demonstrated that all three algorithms are feasible for practical implementation in power system optimization tasks. The computational environment ensured the reliable execution of simulations, and the results were reproducible using similar hardware and software configurations.

### Results and discussion

**Fig.2:**
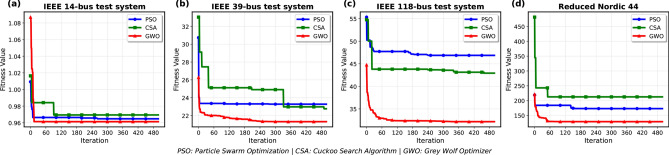
Performance comparison of PSO, CSA, and GWO algorithms across four different power systems: IEEE 14-bus, IEEE 39-bus, IEEE 118-bus, and the Reduced Nordic 44. The objective function value is plotted against the number of iterations. GWO consistently achieves better performance, converging to lower fitness values across all systems.

Figure 2 shows the comparative performance of PSO, CSA, and GWO in these four systems, with fitness value plotted against the number of iterations.

The better performance of GWO across all IEEE test systems can be attributed to its effective balance between exploration and exploitation, as dictated by its hierarchical leadership structure. The algorithm’s ability to dynamically adjust the search agents’ positions based on the alpha, beta, and delta wolves enables it to navigate the search space more efficiently than PSO and CSA.

In smaller systems like the IEEE 14-bus, the performance differences are less pronounced due to the lower complexity of the search space. However, as system size increases, GWO’s advantages become more evident. The higher-dimensional search space of the IEEE 118-bus system presents a greater challenge, where GWO’s mechanisms prevent premature convergence and escape local optima more effectively.

These findings align with theoretical expectations that algorithms with adaptive leadership hierarchies (like GWO) perform better in complex, multi-modal optimization problems. In the Reduced Nordic 44 system, which represents a more complex and interconnected grid, GWO again achieves the best results, converging quickly and reaching the lowest fitness value among the three algorithms. CSA, on the other hand, struggles with higher initial and final fitness values, indicating less efficient optimization in this case. PSO performs moderately well but does not surpass GWO in any of the systems.

This comparison highlights GWO’s better performance, particularly in larger and more complex networks. The results underscore the adaptability and effectiveness of GWO in solving large-scale optimal power flow problems, while PSO offers stable, moderate performance, and CSA tends to lag in efficiency as system complexity grows.

## Optimization in the reduced Nordic 44 model

**Fig. 3:**
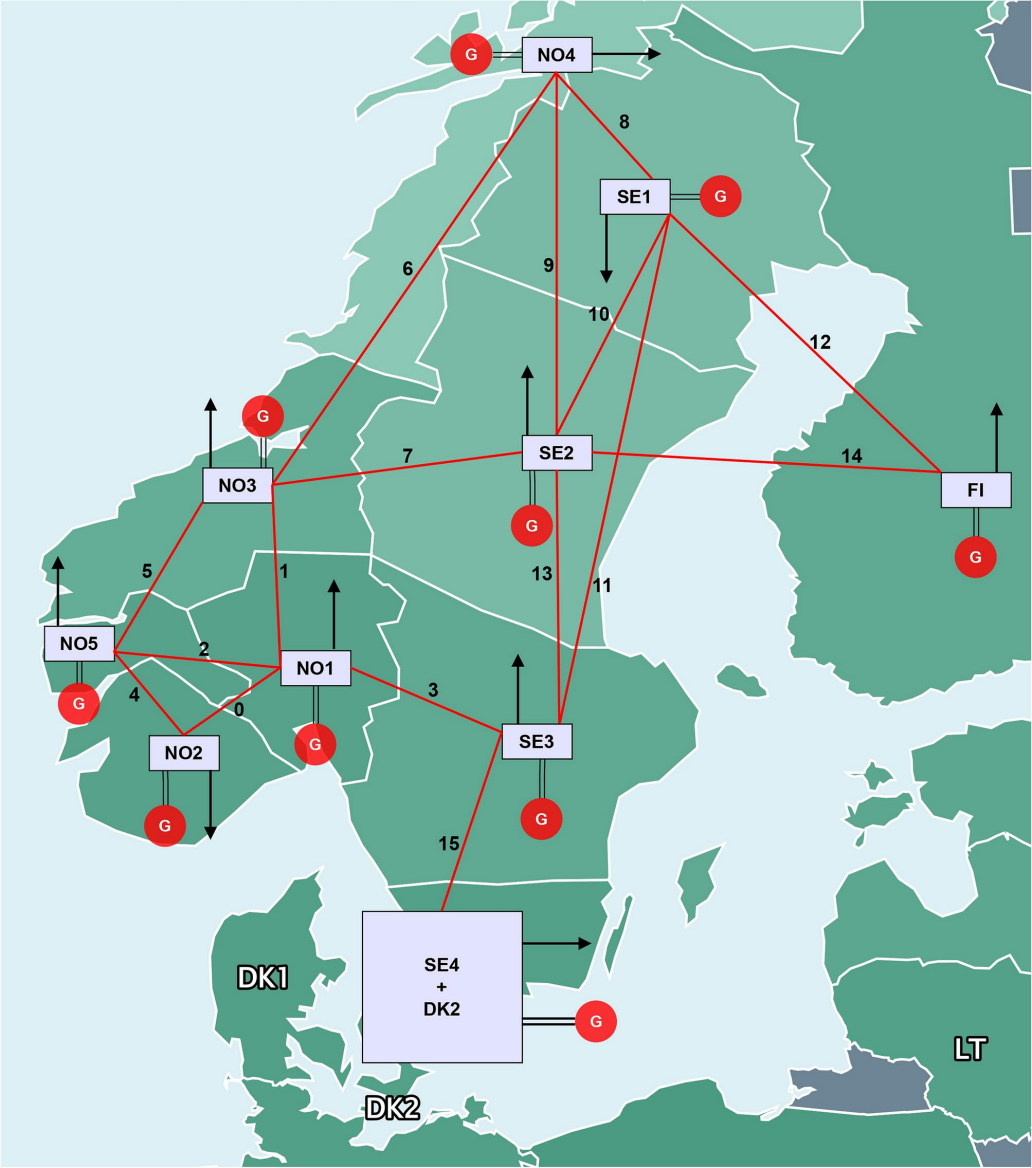
Reduced Nordic 44 model, which is a modified version of the Nordic 44 model, representing a down-scaled version of the future 2030 Nordic 44 model. The line number is reference for Fig. 5.

The Reduced N44 Pricing Area Model used in this study is a simplified and updated version of the Nordic 44 model^52^, reflecting the current and planned network topology of the Norwegian and Swedish power systems. Figure 3 illustrates the modified model, which captures the geographic distribution and interconnections of different pricing areas across the Nordic region. This version has been tailored to accommodate the growing electricity demand, aligned with Statnett’s expansion plans^53^, and to reflect the Nordic pricing zones. Tables 2 and 3 provide the system parameters and line configurations for this reduced model, normalized to a 420kV bus system.

**Table 2:** Reduced Nordic 44 model parameters, under varied load cases, normalized to a 420kV bus system.

Bus	420kV bus system parameters in MW								
	Base case scenario			Light load scenario			Heavy load scenario		
	Generation	Load	Exchange	Generation	Load	Exchange	Generation	Load	Exchange
NO1	2803	7125	0	1299	4750	0	3725	8550	0
NO2	6014	7020	0	2798	4680	−2650	7669	8424	0
NO3	3519	2873	0	1649	1915	0	4132	3447	0
NO4	3854	2625	0	1788	1750	0	5064	3150	0
NO5	10061	4335	0	4666	2890	−700	13261	5202	−1400
SE1	4963	1800	0	4161	1200	0	6418	2040	0
SE2	8400	5475	0	6800	3650	0	8000	6205	0
SE3	12430	13350	0	10287	8900	−2500	14487	15130	−1250
SE4+DK2	2858	6375	0	2303	4250	4000	2595	7225	4000
FI	13000	15586	0	9750	10391	2500	12350	17664	1250

**Table 3:** Line configurations between buses in the reduced Nordic 44 model for 420 kV equivalent lines.

Lines	No. of lines	Line length [km]
NO1–NO2	5x	90
NO1–NO3	1x	200
NO1–NO5	4x	115
NO1–SE3	2x	120
NO2–NO5	5x	100
NO3–NO5	1x	125
NO3–NO4	2x	125
NO3–SE2	1x	50
NO4–SE1	1x	200
NO4–SE2	1x	500
SE1–SE2	3x	210
SE1–SE3	1x	430
SE1–FI	1x	65
SE2–SE3	9x	140
SE2–FI	1x	60
SE3–SE4	6x	130

Three case studies are conducted using the reduced Nordic 44 model to test the proposed approach’s effectiveness in different scenarios. The first case is the base case, which examines the system under normal conditions with no import/export and 75% load. The second case models a light load situation, simulating maximum power export from Norway and Sweden at 50% load. The third case simulates a heavy load case with 90% load and includes import/export. These tests aim to show the method’s robustness in managing power flows between Nordic pricing areas.

**Fig. 4:**
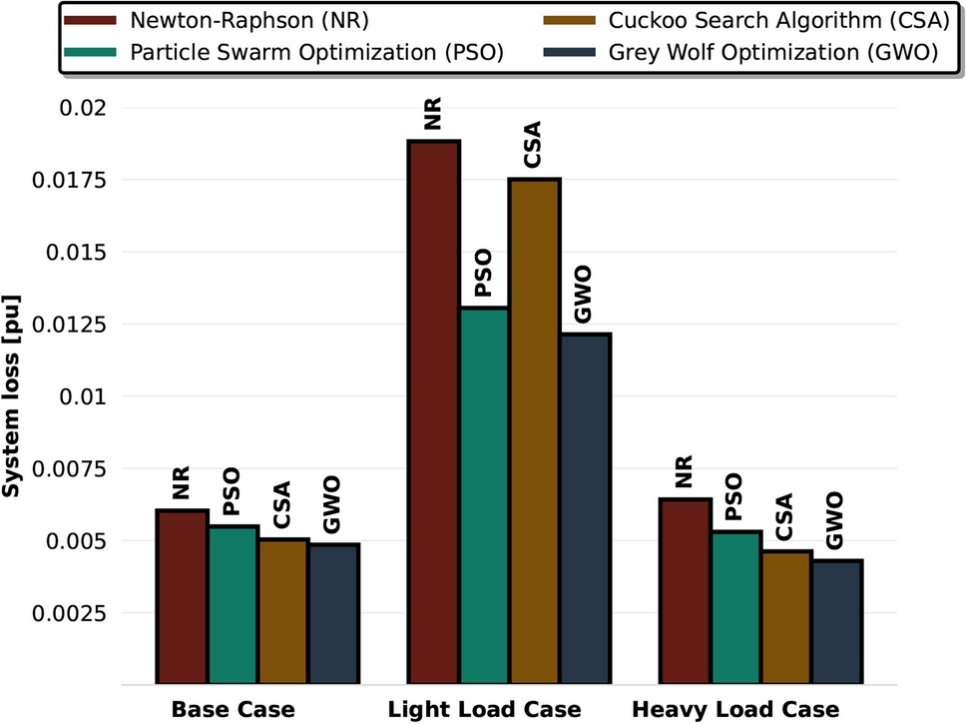
Total system loss in different cases for the reduced Nordic 44 model using different algorithms to reach optimal power flow. All loss values are expressed as a proportion of the total load in the different loading scenarios.

The simulation results are shown in Figure 4, comparing each algorithm’s performance in minimizing power losses. In the base case, all three algorithms-GWO, PSO, and CSA-produced similar results, but GWO outperformed the others, reducing power losses by 11.5% compared to PSO and 3.6% compared to CSA. In the heavy load scenario, GWO also delivered the best performance, reducing losses by 18.9% over PSO and 7.1% over CSA. In the light load scenario, GWO once again showed the lowest power losses, while CSA performed the least out of the three algorithms, reducing losses by only 6.9% compared to the Newton-Raphson method. Overall, these results highlight that GWO is the most effective algorithm for minimizing power losses in the Reduced Nordic 44 model, especially in high-load and high-hydro generation scenarios.

**Fig. 5:**
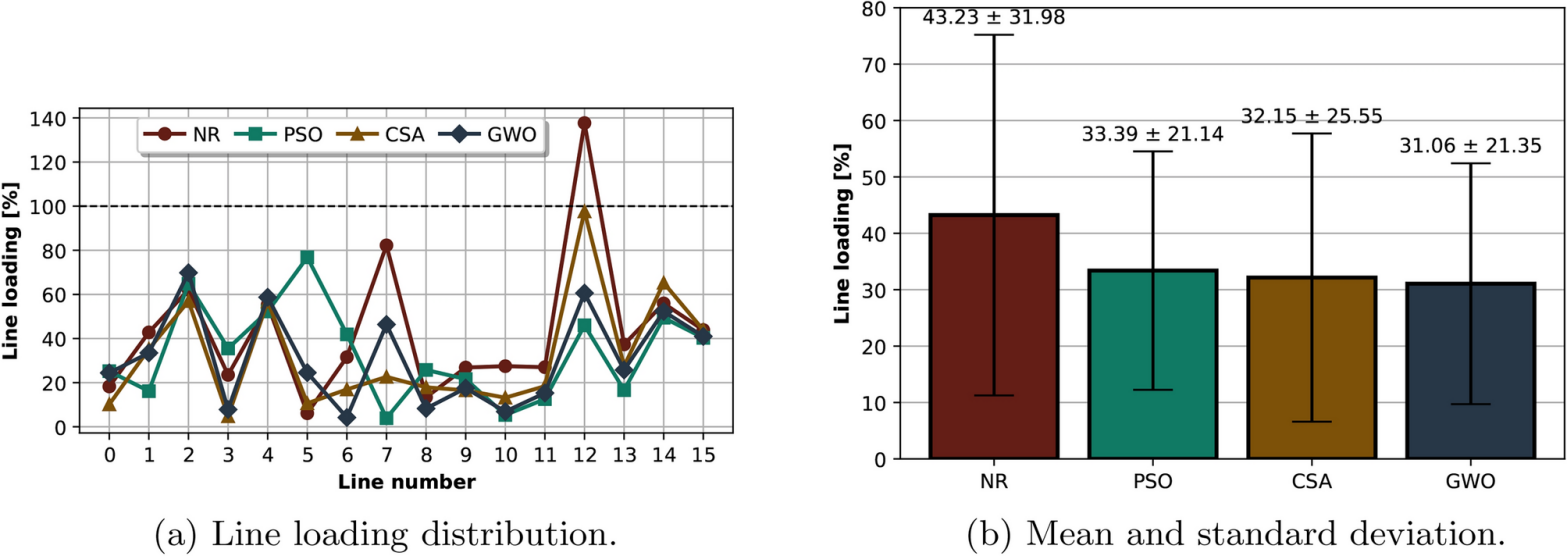
Line loading in the base case scenario for the reduced Nordic 44 model, comparing different metaheuristic algorithms. (**a**) Line loading across different lines for each algorithm. (**b**) Mean and variability of line loading, showing differences in algorithm performance.

Although the primary goal of the optimization is to minimize power losses, it is imperative to understand how this impacts other key factors, such as line loading. Line loading, which measures the amount of current flowing through a transmission line relative to its thermal capacity, plays a critical role in power system analysis. High line loading can lead to transmission line overloads, voltage instability, and potential blackouts. In the base case scenario of the reduced Nordic 44 model, using the traditional Newton-Raphson algorithm resulted in line loading reaching up to 140%, as shown in Figure 5.

This integrated MSO-metaheuristic approach demonstrates significant improvements in line loading distribution as shown in Figure 5 where the line loading dropped significantly to a range between 30% and 100% across the same lines. This reduction highlights a more balanced power distribution, showcasing the effectiveness of these algorithms in handling complex power system optimization challenges. The comparison is further detailed in the subplot, where the mean and standard deviation of line loading for each algorithm are displayed. GWO algorithm stands out, achieving the lowest mean line loading at 31.06%, underscoring its better performance in optimizing power flow. Additionally, GWO recorded a relatively low standard deviation of 21.35, indicating a more uniform and consistent distribution of power across the network.

**Fig. 6:**
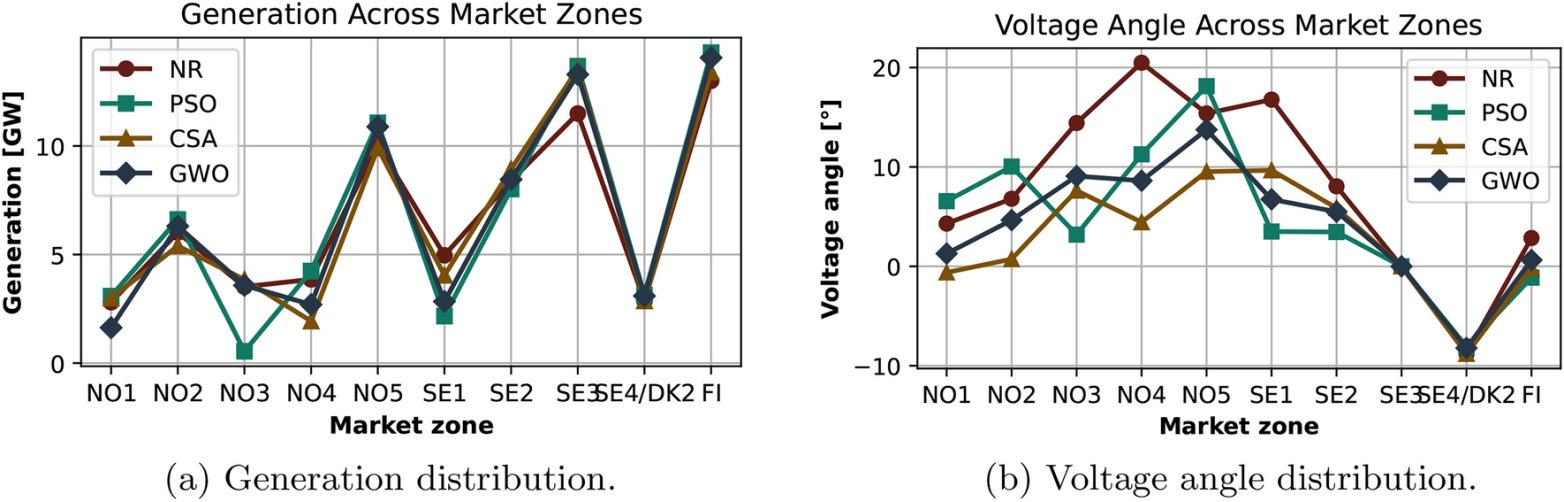
Generation and voltage angle distributions in the base case scenario for the reduced Nordic 44 model. (**a**) Generation distribution across zones for each algorithm. (**b**) Voltage angle across zones, comparing algorithms in minimizing system losses.

Further analysis of generation and voltage angles across different generators offer deeper insights into the effectiveness of the metaheuristic algorithms in the reduced Nordic 44 model, as illustrated in Figure 6. These results demonstrate that the metaheuristic algorithms not only minimize power losses but also optimize generation dispatch and improve voltage profiles across the network. The holistic improvement in both active power generation and voltage angles underscores the effectiveness of these algorithms in enhancing the operational performance of complex power systems.

The use of metaheuristic algorithms resulted in increased generation at the slack bus SE3 compared to the NR method, enhancing overall system efficiency and stability. These algorithms dynamically adjust power generation, aiming to achieve a global balance, although individual generator outputs may vary. This adaptability is a key strength of the algorithms, though it does not consistently guarantee better performance. Additionally, the voltage angle, which represents the phase difference between the generator and load bus voltages and is vital for power flow and system stability, decreased when using metaheuristic methods. This reduction demonstrates the algorithms’ ability to fine-tune power flow and enhance system stability, leading to improved overall performance.

The superior performance of GWO across different scenarios can be attributed to its hierarchical leadership structure and adaptive search mechanism. Unlike PSO, which relies on inertia weight and acceleration coefficients that remain relatively static throughout the optimization process, GWO dynamically adjusts its exploration and exploitation phases through the influence of alpha, beta, and delta wolves. This adaptability is particularly valuable in the heavy load scenario, where system constraints become more binding and the solution space more complex. The correlation between algorithm performance and system loading also reveals important insights-GWO’s advantage becomes more pronounced as system loading increases, suggesting that its search strategy is especially effective in more constrained conditions. Furthermore, the consistent performance across different network configurations demonstrates the algorithm’s robustness to topological variations, which is an important consideration for practical implementation in diverse power systems.

The study reveals that the GWO algorithm is particularly effective in minimizing power losses in the reduced Nordic 44 model, especially under high load and hydroelectric generation conditions. However, it is important to note that these results are specific to the study’s context. In reality, the Nordic grid heavily relies on market clearing mechanisms for power dispatch. Therefore, further research is needed to assess the practical application of these algorithms in real-world settings. A significant challenge in real-time operations is the scalability of these algorithms, as decisions must be made rapidly. Integrating these metaheuristic methods into existing grid management systems, which often rely on traditional algorithms like Newton-Raphson, presents additional obstacles. Implementing such advanced techniques for real-time decision-making would require updates to existing software, workforce training, and possibly even changes to regulatory frameworks.

## Multiple slack bus operation framework

The idea of a slack bus, also known as a swing bus, serves as the foundation for power system dynamics. It balances the network by adjusting power output, ensuring total generation equals the load plus system losses^54^. While traditionally a single bus acted as the slack bus to counteract power imbalances, real-world systems distribute this responsibility across several dispersed generators. This shared approach, MSO, especially in large systems like the reduced Nordic 44 model, leads to balanced operations and enhances system reliability. Given the intricacies of power flow in extensive systems, multiple slack buses better reflect actual operational conditions, refining power flow study precision^55^.

**Fig. 7:**
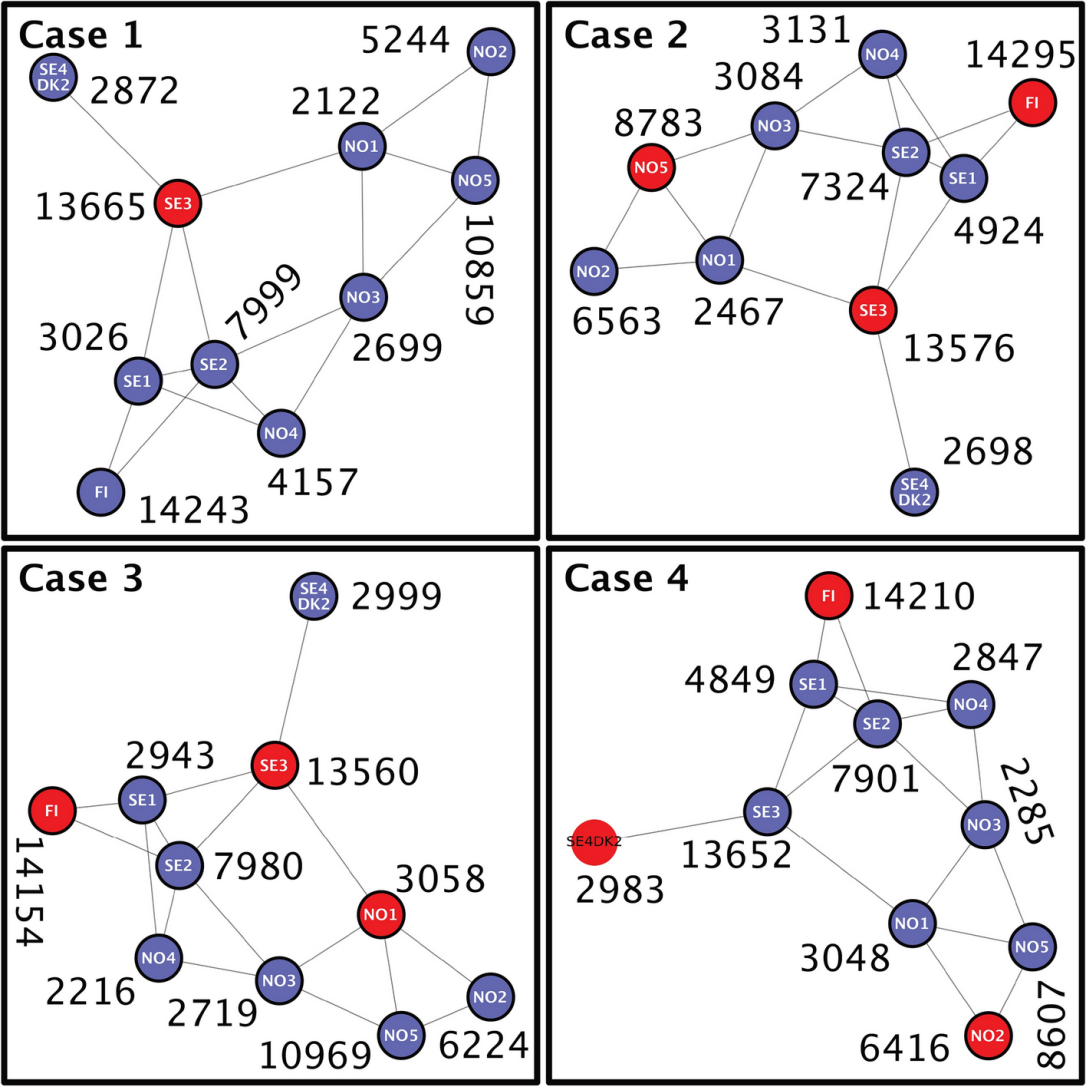
Four slack bus scenarios in the reduced Nordic 44 model with generation magnitudes given in MW.

In the reduced Nordic 44 model, four unique scenarios of slack bus operation were analyzed as depicted in Fig. 7 and Table 4.

**Table 4:** Overview of different strategies for slack bus allocation in various operational scenarios.

Case	Slack Bus	Description
1	SE3	SE3 as the sole slack bus, the primary control node representing the Stockholm region. A major consumption center with strong international connections.
2	SE3 NO5 FI	Distributed control with slack buses at the largest generators in Sweden, Norway, and Finland. Major power producers that share control of power flow.
3	NO1 SE3 FI	Control centered around major load points, with slack buses in major consumption centers in Norway, Sweden, and Finland.
4	NO2 SE4+DK2 FI	Slack buses based on international interconnections, focusing on power transaction control and influenced by inter-country power transactions.

The first case, Single Slack Operation, appoints SE3 as the sole slack bus, serving as the primary control node for the system. SE3 represents the Stockholm region in Sweden and is a major consumption center with strong connections to neighboring countries, therefore it’s a strategic point for coordinating the balance between power production and consumption across the network. The second case, MSO (SE3+NO5+FI), explores a more distributed control configuration. Here, the largest generators in Sweden, Norway, and Finland, specifically SE3, NO5, and FI, function as slack buses. This case models a situation where the control of power flow is shared among the major power producers. The third case, MSO (NO1+SE3+FI), proposes the major load points in the three countries as the slack buses. For instance, NO1 represents the Oslo region in Norway, a major consumption center with a strong connection to Sweden. This scenario examines the power flow management when control is centered around major demand nodes. Lastly, the fourth case, MSO (NO2+SE4DK2+FI), bases the selection of slack buses on their interconnection to other countries, providing a scenario where power flow control is influenced by inter-country power transactions. NO2 represents the Bergen region in Norway, which has connections to the UK and the Netherlands, and SE4 represents the Malmo region in Sweden, connected to Denmark and Germany.

Following the establishment of the slack bus configurations in each scenario, an essential aspect was to determine the slack weights for each chosen slack bus which is their participation factor as explained in Section 3 and determined using equation (8). Buses with larger generation capacities handle more imbalance, optimizing resource use and reducing potential generator overloads.

**Fig. 8:**
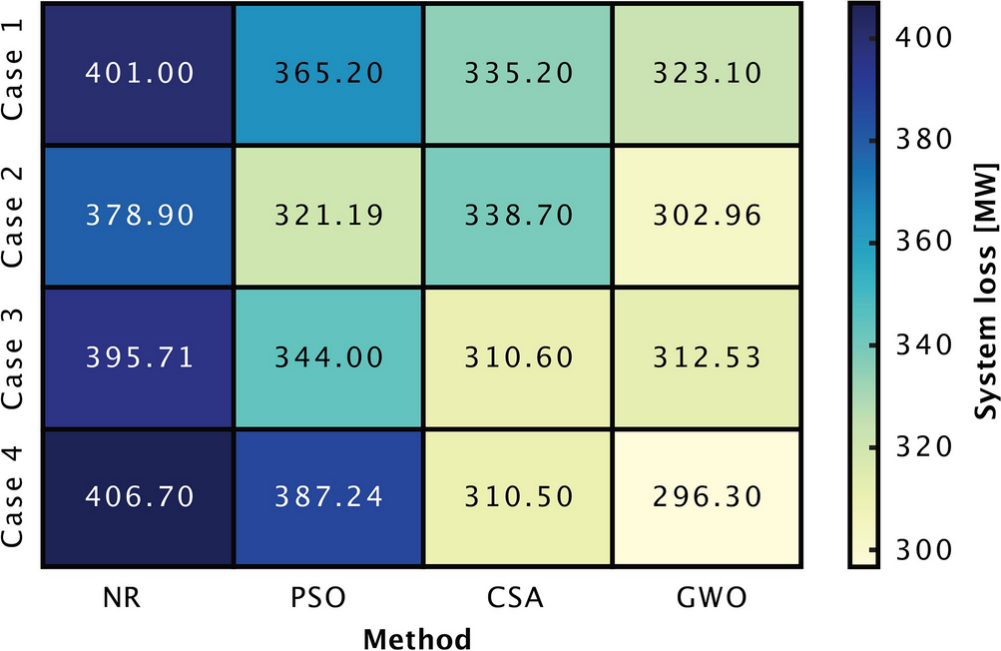
System loss heatmap comparing the different cases of slack bus scenarios, emphasizing the efficiency of multi-slack operations and metaheuristics in reducing transmission losses in all cases.

Fig. 8 shows a heatmap comparing the system losses across four distinct slack bus configurations using the traditional NR method and the metaheuristic optimization technique. Intriguingly, even without the application of GWO, it is observed that MSO itself aids in reducing system losses. Specifically, the system losses under the NR method decrease by 5.7% in Case 2 (SE3+NO5+FI) compared to single slack bus operation, suggesting the inherent benefits of MSO in improving system efficiency.

The analysis of system losses in different slack bus configurations, as presented in the heatmap, provides both a general and a detailed perspective. The horizontal analysis, which is based on rows that correspond to various slack bus configurations, shows a consistent pattern: the GWO algorithm always shows the lightest shade, demonstrating its effectiveness in minimizing system loss. The substantial reduction in system losses achieved by GWO indicates that advanced optimization techniques can enhance the efficiency of large-scale power systems. This observation suggests that when the slack bus configuration focuses on nodes with significant inter-country power exchange, and when optimized using the GWO approach, the power system trends towards its most efficient operational state, consequently achieving minimized system losses.

GWO’s consistent effectiveness is once again highlighted in a vertical perspective that explores the specific performance of each algorithm in various settings. The strong performance of GWO is emphasized by its continually lighter colors. PSO and CSA also showcase their robustness, with peak performance variations of 17.1% and 7.3% respectively across their best and worst scenarios, while GWO’s efficiency range stands at approximately 8.3%. Notably, GWO’s optimization in Case 4 results in a minimum system loss, further highlighting the synergy of strategic slack bus configurations and the GWO approach. Collectively, while each algorithm has its merits, GWO emerges as the most efficacious in steering power systems to their optimal operational thresholds.

The consistent performance of GWO in scenarios with high load and hydroelectric generation suggests its suitability for systems with similar characteristics. By effectively minimizing power losses and optimizing line loading, the algorithm contributes to improved system stability and reliability. Moreover, the reduction in voltage angle differences implies better synchronization across the network, which is critical for maintaining system integrity. The ability of metaheuristic algorithms to adjust generator outputs dynamically highlights their potential to support grid operations under varying conditions.

When compared to recent relevant studies, this integrated MSO-metaheuristic approach offers several advantages. While previous works such as^21,22^ have explored distributed slack bus formulations and^10,34^ have applied various metaheuristic techniques to OPF problems, this research uniquely combines these approaches with quantifiable benefits. Unlike^28^, which focused only on slack bus selection effects, and^35^, which applied metaheuristics without the MSO framework, this comprehensive approach demonstrates significant performance improvements across multiple metrics. The 5% system loss reduction from MSO implementation alone, with an additional 8.3% decrease when combined with GWO, surpasses the improvements reported in similar studies. Furthermore, this analysis of different slack bus configurations provides practical insights not available in existing literature, particularly for complex interconnected systems like the Nordic grid.

These results suggest that integrating metaheuristic algorithms with MSO can aid in addressing challenges related to renewable energy integration, demand variability, and cross-border power exchanges, which are prevalent in modern power systems.

## Conclusion

This paper presents a novel integration of metaheuristic techniques with MSO framework, addressing a significant gap in power flow optimization research. By combining these previously separate approaches, the study demonstrates substantial improvements in system efficiency beyond what either approach achieves independently. Metaheuristic strategies are known to effectively reduce power loss and elevate the system’s efficiency. The findings of this study are as follows:

1. The GWO method offered better performance in terms of overall system loss minimization with its 11.5% and 3.6% further loss reduction than PSO and CSA, respectively, in the base case of the reduced Nordic 44 model. PSO demonstrated better performance compared to CSA.
2. Multiple slack bus operations in the reduced Nordic 44 model led to a 5.7% system loss reduction compared to single slack bus operations. Pairing this with metaheuristics can further decrease system losses by 13–27%. The selection of the right slack bus, considering geographic factors and load generation dynamics, is crucial.

In extensive systems, GWO consistently shows better performance compared to the other algorithms, highlighting its robustness in managing complexity. However, in smaller systems, the performance deviation between GWO, CSA, and PSO narrows significantly. Across these variances, the strategic implementation of MSO with metaheuristics consistently achieves optimal system loss reduction.

It is also important to understand that while these algorithms have distinct advantages in certain situations, potential redundancies or inefficiencies might emerge depending on specific operational conditions, necessitating further investigation. The work is limited by the use of simplified models and specific operational scenarios, which may not capture all the complexities of real-world power systems. Additionally, while the model considers various load conditions, it does not fully account for the stochastic nature of renewable energy sources, which represents an important consideration for future work. Also, the practical implementation of these algorithms in real-time operations requires consideration of computational efficiency and integration with existing grid management systems.

Future research could extend this work in several promising directions. Incorporating the uncertainties of renewable energy sources into the MSO framework would increase its applicability in modern power systems with high renewable penetration. Likewise, investigating adaptive participation factor determination methods could further enhance the effectiveness of the MSO framework in real-world applications. In addition to the MSO framework and metaheuristic optimization, demand response (DR) offers complementary approaches to enhance grid efficiency. As demonstrated by^56^, relaying-assisted communications for DR can significantly improve system performance through coordinated consumer participation. Integrating this MSO-metaheuristic approach with demand response mechanisms could provide additional flexibility for managing power flows in systems with high renewable penetration, representing a promising direction for future research.

## Data Availability

The data are within the article.

## List of symbols

*F*(*x*, *u*): Optimization objective function
*g*(*x*, *u*): Equality constraints
*h*(*x*, *u*): Inequality constraints
*x*: Control variables
*u*: State variables
$P_i^G$: Active power generation at bus *i*
$Q_i^G$: Reactive power generation at bus *i*
$P_i^L$: Active power load demand at bus *i*
$Q_i^L$: Reactive power load demand at bus *i*
$V_i$: Voltage magnitude at bus *i*
$\delta _i$: Voltage angle at bus *i*
$G_{ik}$: Conductance between bus *i* and *k*
$B_{ik}$: Susceptance between bus *i* and *k*
$P_{\text {loss}}$: Total system active power loss
*NL*: Number of transmission lines in the system
$K_i$: Participation factor for generator *i*
$\Delta P_i^G$: Additional power that generator *i* needs to produce
$x_i^k$: Position of particle *i* at iteration *k*
$v_i^k$: Velocity of particle *i* at iteration *k*
$x_i^{\text {lbest}}$: Local best position of particle *i*
$x^{\text {gbest}}$: Global best position in the swarm
$\alpha _i, \beta _i$: Random numbers between 0 and 1
$X_{\alpha }, X_{\beta }, X_{\delta }$: Positions of alpha, beta, and delta wolves
$D_{\alpha }, D_{\beta }, D_{\delta }$: Distances to alpha, beta, and delta wolves

## Abbreviations

AGC: Automatic Generation Control
ANN: Artificial Neural Network
CSA: Cuckoo Search Algorithm
GWO: Grey Wolf Optimization
MSO: Multiple Slack Bus Operation
NFL: No Free Lunch
NR: Newton-Raphson
OPF: Optimal Power Flow
PSO: Particle Swarm Optimization
SE: Sweden
NO: Norway
FI: Finland
DK: Denmark
